# Effect of coercive measures on treatment outcome in involuntarily admitted patients in Amsterdam

**DOI:** 10.3389/fpsyt.2023.1240129

**Published:** 2023-09-22

**Authors:** L. F. M. van der Post, K. J. Nusselder, J. Peen, U. Nabitz, J. M. Dekker

**Affiliations:** ^1^Research Department, ARKIN Mental Health Care, Amsterdam, Netherlands; ^2^Department of Psychology, Faculty of Psychology and Pedagogy, Vrije Universiteit, Amsterdam, Netherlands

**Keywords:** involuntary admission, coercion, restraint, seclusion, forced medication, psychiatry, inpatient, effect

## Abstract

**Objective:**

The prevalence of involuntary admissions rose the last forty years in European countries, including the Netherlands. Involuntary admissions result in seclusion, physical restraint and forced medication in approximately 40% of patients. We looked at whether treatment outcomes differ in patients with and without coercive measures.

**Methods:**

Using The Health of the Nation Outcome Scales (HoNOS) to measure treatment outcomes, we studied the files of 786 patients admitted involuntarily to an Amsterdam clinic. We applied Generalised Linear Models to determine whether the use, or not, of coercive measures during treatment was associated with a difference in outcomes.

**Results:**

19% of the cohort were secluded in a High Security Room (HSR); 24% were secluded in their own room and/or received forced medication. After adjustment for the influence of diagnosis, disorder severity (initial HoNOS score) and treatment duration, the HSR group had, on average, a HoNOS difference score that was 2.4 points lower than patients without coercive measures (CI −4.0 to −0.8.; *p* 0.003). In the *seclusion in own room* group, this score was 2.6 points lower (CI −4.0 to −1.1; *p* 0.001), corresponding to an effect size of 0.35 and 0.40, respectively.

**Conclusion:**

Seclusion, whether or not in combination with forced medication, was applied to two-fifths of patients. The HoNOS scores of the group without coercion improved by nearly two and a half points more on average than those of the two groups with coercion. A causal relationship between coercion and treatment outcome could neither be confirmed nor excluded on the basis of our results.

## Introduction

In the closing decades of the last century, the prevalence of involuntary admissions rose in several European countries (1). That rise continued in the Netherlands between 2003 and 2017 (2).^1^

Involuntary admissions result in seclusion, physical restraint and forced medication more often than voluntary admissions (3–6). The 2010 Eunomia study conducted in ten European countries (not including Netherlands) studying the use of coercive measures during psychiatric admissions found that an average of 40% of admitted patients, ranging from 20% (Spain) and 59% (Poland), were subjected to coercive measures (5). Forced medication was used most frequently, followed by physical restraint, and finally seclusion. Forced medication is defined as: *activities using restraint or strong psychological pressure (involving at least three staff members) to administer medication against the patient’s will.* Seclusion refers to: *the involuntary placement of an individual locked in a room alone, which may be set up especially for this purpose.* Mechanical restraint is defined as: *fixing at least one of the patient’s limbs with a mechanical device or being held by a staff member for longer than 15 min* (5).

A 2015 review article, which compared the use of coercive measures in the Netherlands with 14 other countries, made it clear that in the Netherlands, coercion mainly constituted of seclusion, while in Germany, coercion was mostly mechanical restraint (7). The authors state that this difference seems to be based on tradition rather than on results of research.

The risk factors for coercive measures were gender (more in men), diagnosis (more in schizophrenia) and severity of psychopathology upon admission (the more severe the pathology, the more seclusion) (5).

Although coercive measures are therefore widely used in psychiatry, a 2019 review of 53 articles – most of them from Europe and the United States – states that *conclusions on protective or therapeutic effects of seclusion and restraint are more difficult to draw. Our results provide little evidence for these outcomes* (8).

Van Melle et al. put it more straightforwardly: *effects on reducing stimuli and creating a context for calming the patient, which are often mentioned as a reason for secluding an agitated patient, have not been demonstrated* (9).

### Research question

Given the limited scientific evidence for the effectiveness of coercive measures, we studied the files of psychiatric patients who were admitted involuntarily to an Amsterdam clinic over a five-year period. We addressed the following research questions:

1. Which types of coercive measures were used during the given period?
2. Are there differences upon admission between the socio-demographic and clinical characteristics of patients who were, or were not, subjected to coercive measures?
3. Is there a difference in treatment outcome between patients who underwent coercive measures (and different forms of those measures) and patients without coercive measures after adjustment for the influence of diagnosis and disorder severity?

## Method

### Setting

The psychiatric clinic in this study is located in the centre of Amsterdam and provides short-term clinical care for people in acute psychiatric crisis. Most admissions are involuntary and they involve patients in a potentially dangerous crisis situation due to a mental disorder, mostly in combination with social dysfunction and substance abuse (10). The average stay varies from 4 days to 4 months. There are 90 clinical 24/7 psychiatric beds, alongside a range of part-time treatments for about 30 patients. A previous publication by our research group described this psychiatric clinic and its treatment programmes in detail (10).

### Patients

This study looks at 2610 patients who, between 1-1-2013 and 31-12-2017, completed their treatment at the clinic. Involuntarily admitted patients accounted for 61% of the total patient group (*N* = 1,597). These were emergency admissions for patients with Severe Mental Illness (SMI) (11). These SMI patients have complex psychiatric problems with a high level of comorbidity (somatic and substance use) and significant social problems in terms of housing, lack of social support, finances and nuisance behaviour. They also consume considerably more care than patients with milder disorders (12). Of the 1,597 patients involuntary admitted, 786 underwent initial and final assessments with the Health of the Nation Outcome Scales (HoNOS). These 786 patients constituted the research cohort for this study.

### Patient characteristics

The following patient characteristics were recorded when treatment began: age, gender, civil status, cultural origin, last completed education, source of income, country of birth, nationality and main psychiatric diagnosis according to DSM-IV-TR broken down into four categories: *psychotic disorder*, *depressive disorder*, *other disorders* and *no psychiatric disorder or psychosocial problems*. Scores were missing for educational level, source of income and civil status in more than 50% of the cohort and these characteristics were therefore excluded from the analyses.

### Registration of coercive measures

Data on coercive measures were collected using the Argus rating scale (13). The scale includes three types of coercive measures: seclusion, physical restraint and forced medication (13). Seclusion was defined as the seclusion of a patient in a High Security Room (HSR) or in the patient’s own room. A HSR has been built and equipped with limited, adapted furniture in accordance with safety standards set by the Dutch Mental Health Inspectorate. Given that, generally speaking, Dutch patients and treatment staff regard confinement in own room, –with normal furniture and other equipment–, as a less invasive measure than confinement in an HSR, these two types of confinement were registered separately.

### Initial severity of illness and general improvement during admission

The Health of the Nation Outcome Scales (HoNOS) change score was used to assess initial severity of illness and treatment response and was determined at admission and discharge (10). In the Netherlands, the HoNOS is used to evaluate the severity of psychiatric, social and physical problems in schizophrenia and other psychiatric disorders in the context of Routine Outcome Assessment (ROM) (14). HoNOS includes 12 scales (aggression; non-accidental self-injury; problem-drinking or drug-taking; cognitive problems; physical illness or disability; hallucinations and delusions; depressed mood; other mental and/or behavioural problems; problems with relationships; problems with activities of daily living; problems with living situation; and problems with occupation and activities). Each scale can be rated on a 5-point scale from 0 (no problem) to 4 (severe/very severe problems). The HoNOS looks at illness severity, overall clinical pre-post change, overall symptom improvement and length of hospital stay (15).

The reliability of the HoNOS was found to be fair to good. The validity was considered to be satisfactory for the following reasons: item patterns of various syndromes differed in the expected manner, total scores varied according to treatment intensities, and the HoNOS related fairly well to other scales Inter-rater agreement was also satisfactory to good for most items, except for items 2 (self-injury), 8 (psychiatry/other behaviour), and 10 (activities of daily living) (14).

The difference between the initial and final HoNOS scores was the outcome measure in our study. Negative differences reflect a higher HoNOS score at the end of treatment compared with the initial score and therefore a negative treatment response. Positive scores reflect the opposite: a positive treatment response. The clinical assessment of the HoNOS is included in the clinic’s intake and discharge protocol and is part of the Routine Outcome Assessment (14, 15). Upon admission, doctors training as psychiatrists make the assessment and, upon discharge, this is done by the nurses on the ward who have closely monitored the patient’s functioning.

### Statistical analyses

We checked the study cohort of 786 patients with an initial and final HoNOS measurement to see if it was representative for the entire group of patients admitted under a BOPZ (*N* = 1,597) measure by comparing the sociodemographic and clinical characteristics of the study cohort with those patients with missing or incomplete HoNOS measurements.

Generalised linear models (GENLIN) were used to determine whether the use, or not, of coercive measures was associated with the mean difference between the initial and final HoNOS measurements. This involved controlling for the influence of the other independent variables.

The GENLIN analysis followed a stepwise procedure to make a selection of independent variables associated with the outcome measure. The variables were then entered in a preliminary model, after which variables with no effect, or a very small effect (parameter: likelihood Ratio Chi-Square), were removed from the model (16).

SPSS 19 (17) was used for statistical analysis.

### Data protection and medical ethics review

During analysis and reporting, patient privacy was protected in accordance with legislation and regulations applicable in the Netherlands. The analyses of the outcome of Routine Outcome Assessment (ROM) are expected to contribute to improving the quality of care. Research of this kind is not covered by the Dutch Medical Research Involving Human Subjects Act (Dutch acronym: WMO). A medical ethics review was therefore not required.

## Results

### Is the study cohort a representative sample of all patients admitted involuntarily during the study period?

We compared the study cohort (*N* = 786) with an initial and final HoNOS score with the group of patients with no, or only an initial, score (*N* = 811), looking at the following characteristics: gender, age, main Axis I diagnostic category DSM IV TR, length of stay and initial severity of illness (initial HoNOS score). No significant differences were found between the two groups in terms of gender, cultural origin and initial mean HoNOS score (Chi^2^ *p* > 0.05). By contrast, the mean length of stay in patients without a complete measurement was significantly shorter than in the study cohort: 44.5 (SD 48.2) and 64.8 days (SD 5.9; *p* < 0.001) respectively. The group without a complete measurement was also slightly younger: 41.9 (SD 12.5) as opposed to 43.2 years (SD 12.0; *p* = 0.007). There was also a minor difference in the distribution of the diagnostic categories: the group without a complete HoNOS assessment included slightly more patients (72% vs. 68%) with schizophrenia and other psychotic disorders, and slightly fewer patients with a mood disorder.

### Which coercive measures were used?

Table 1 shows the incidence of three forms of coercive measures: forced medication, seclusion in patient’s own room and seclusion in a HSR. Other forms of physical restraint like mechanical restraint did not occur in this clinic. Of the 341 patients (43% of study cohort) who underwent restraints, 152 (19%) were secluded in a HSR (at least once), with or without forced medication. Some patients in this group (113;14%) were also secluded in their own room at other moments. Forced medication without either form of seclusion was used in 65 (8%) patients. On the basis of this distribution of coercive measures, we divided the patients who underwent coercion into two groups. The first group (the HSR group) consisted of patients who underwent seclusion in a HSR with or without forced medication (at least once during treatment), the second of patients who were secluded in own room and/or received forced medication (the own room group). The coercive measures for the latter group were considered to be less drastic.

**Table 1:** Incidence of coercive measures for study cohort (*N* = 786).

	*N*	%
No coercion	445	57
Forced medication only	65	8
Seclusion in patient’s own room only	70	9
Forced medicatien and seclusion in patient’s own room	54	7
Seclusion in HSR only	15	2
Forced medication and seclusion in HSR	24	3
Seclusion in both HSR and own room	42	5
Forced medication and both types of seclusion	71	9
Total	786	100

### Characteristics of the study cohort by coercive measures

The two groups of patients who underwent coercive measures during admission did not differ significantly from the group without coercion in terms of mean age, cultural background, distribution of major diagnostic categories and mean HoNOS score at end of treatment (α 0.007; Table 2).

**Table 2:** Socio-demographic and clinical characterististics of study cohort: patients who underwent coercive measures during treatment and patients who did not (*N* = 786).

		Seclusion in HSR with or without forced medication	Seclusion in own room and/or forced medication	No coercive measures	P^c^
Total (*N*)		152	189	445	
Gender	*F* (%)^a^	36	54	43	0.002
Mean age (SD)		40	46	43	0.009^d^
Cultural background	Netherlands (%)	22	22	25	0.717
	Other Europe (%)	10	13	10	
	Surinam and Dutch Caribbean (%)	17	10	14	
	Africa Incl. North-Africa (%)	10	11	11	
	Other non-Dutch^b^ (%)	41	44	41	
Main diagnostic category DSM IV TR	Schizophrenia and other psychotic disorders(%)	63	75	67	0.020
	Mood disorders (%)	29	21	23	
	Other disorders (%)	9	4	10	
Mean length of treatment in days (SD)		87 (50)	87 (54)	59 (45)	<0.001^e^
Mean HoNos score start treatment (SD)		19.2 (6.7)	19.3 (6.5)	17.4 (6.5)	0.003^d^
Mean HoNos score end treatment (SD)		10.8 (9.5)	10.7 (8.9)	8.7 (8.0)	0.008^d^

a Column percentage.

b Including 33% missing cultural background.

c Significance level after Bonferroni correction 0.007.

d Seclusion in HSR compared with no coercive measures; independent samples *T*-test.

e Mann Whitney U-test.

Women were subjected to coercive measures less often than men: 36% as opposed to 42%. Men were also secluded in a HSR significantly more often (64%), while women were secluded in their own room and/or received forced medication only more often (54%) (*p* 0.002).

Of the patients from the *HSR group*, 41% were admitted for more than three months. This percentage was 40% in the *own room* group and 19% in the group without coercion (Chi^2^ *p* < 0.001). The mean stay was 87 days for both coercive measures groups (SD 50 and 54 respectively), and 59.3 for the no coercive measures group (SD 45.3; *p* < 0.001). Both groups of patients who received coercive treatment had a higher initial HoNOS score than the group that did not: 19.2 (SD 6.7 and 6.5 respectively) as opposed to 17.4 (SD 6.7; *p* 0.003).

### Treatment outcome for both HSR and own room group by comparison with patients without coercion, controlled for the influence of sociodemographic and clinical characteristics, as well as treatment duration

In 71% of all patients in the study cohort of involuntary admitted patients, psychiatric symptoms (measured with the HoNOS) were reduced. In 19% they remained the same and in 10% they increased during treatment.

Generalised linear models were used to investigate the relationship between the presence or absence of coercive measures and treatment outcome, adjusted for the influence of the co-variables that were significantly associated with the outcome measure (Table 3). In this analysis, we compared the group that underwent seclusion in a HSR and the own room group with the group without any coercive measures (*N* = 526).

**Table 3:** Difference in treatment outcome for patients with seclusion in HSR or seclusion in own room (reference: no coercive measures), controlling for the influence of co-variables; GENLIN (*N* = 786).

		*B*	Interval		*P*
			lower	upper	
Coercive measures	Seclusion in HSR with or without forced medication	−2.417	−4.003	−0.831	0.003
	Seclusion in own room and/or forced medication	−2.618	−4.097	−1.139	0.001
	No coercive measures	1			
Main diagnostic category DSM IV TR	Schizophrenia & other psychotic disorders	2.822	0.672	4.972	0.010
	Mood disorders	4.985	2.603	7.367	<0.001
	Other disorders	1			
Total score HoNOS start treatment		1.924	1.643	2.206	<0.001
Length of treatment (days)		0.042	0.030	0.054	<0.001
*Omnibustest model fit*		Likelihood Ratio Chi2		df	*P*
Model		213.359		6	<0.001

Adjusted for the influence of diagnostic category, initial HoNOS score and duration of treatment (in days), patients who underwent seclusion in a HSR seemed to have, on average, a HoNOS difference score that was 2.4 points lower than patients without coercive measures (CI −4.003 to −0.831; *p* 0.003). For the seclusion in own room and/or forced medication group, this score was 2.618 points lower (CI 4.097—1.139; *p* 0.001). These mean differences correspond with effect sizes (Cohen’s d) of 0.35 and 0.40, respectively.

In addition to main diagnostic category, initial HoNOS score and length of treatment, we also added the other co-variables to the model. However, these co-variables did not contribute to the model fit and were excluded from the prediction model.

## Discussion

### Key findings

More than two-fifths of the involuntarily admitted patients were subjected to coercive measures. For about one-fifth of the study cohort, this meant seclusion in a HSR with or without forced medication. Another fifth received forced medication only and/or separation in own room. In nearly 71% of all patients, psychiatric symptoms (measured with the HoNOS) were reduced. In 19% they remained the same and in 10% they increased. The initial HoNOS scores of patients with coercive measures were about two points higher on average and they therefore had more severe pathology on average than patients with no coercive measures. Treatment duration for these patients was also about one month longer on average.

Adjusted for influence of diagnostic category, level of initial HoNOS score and admission duration, the two groups with coercion had improvements in HoNOS scores that were nearly two and a half points lower on average than the group without coercion, a difference with a small effect size.

### Findings compared with results of previous research

With regard to the 71% of patients whose symptoms decrease during treatment, it is important to also look at the scores of the subscales. A previous publication by our research group based on the same data showed that the patients improved most in regard to the subscales for psychotic problems, aggressiveness and social problems (10).

The nature of the coercion used in our study cohort differs from the practice in other European countries (5). The difference with Germany – where mechanical restraint was the most common coercive measure (and not seclusion) – is particularly pronounced (7).

It was to be expected that patients subjected to coercive measures would have more severe pathology at the start of treatment and this confirms the results of previous research (5, 6, 18). The longer stays in this group also match the conclusions of previous research (19–21).

Previous research also found that male gender was also a risk factor for the use of coercive measures (5, 18). Our data confirm this, while men were also secluded in a HSR significantly more often than women.

Our finding that patients without coercion had slightly better treatment outcomes than those with coercion is not easy to compare with previous results. There was no study with a similar design and dataset in the 2019 review of 53 articles (8).

However, a causal relationship between the use of coercion and less favourable treatment outcome can neither be concluded nor ruled out on the basis of our results. It can be supposed that the patients with coercion at the outset of their treatment differed from those without coercion in terms of characteristics that were not available in our data. In particular, there is the willingness to take medication as advised and also to follow other treatment advice (in other words, compliance). We can assume that coercion was used in patients who were unwilling or reluctant to follow treatment advice, a characteristic that the HoNOS does not measure. It is reasonable to expect that patients with low compliance – regardless of the influence of the use of coercion – will have poorer treatment outcomes than patients with good compliance. Recent research confirmed the relationship between low compliance and the likelihood of seclusion (22).

### Study limitations

There are variables missing from our dataset that could influence treatment outcome. This is the case, for example, for the level of compliance, but also for variables that, in turn, may affect the level of compliance and therefore the need to apply coercive measures. These include negative experiences with previous psychiatric (and possibly coercive) treatment and the level of social support available to the patient. Both variables were found to influence the level of compliance and therefore the need for coercive treatment (22).

Another limitation is that the HoNOS measures psychiatric symptomatology, but not other possible effects of treatment such as improvement in compliance or improvement in social functioning.

### Suggestions for further research

The central question is whether the difference found in treatment outcome persists when controlling for the influence of non-compliance and negative experiences with previous psychiatric treatment and the level of social support available to the patient. This question has to be addressed in new research. If the difference in treatment outcome no longer exists after adjustment for these two variables, the question of the causal relationship between coercive measures and treatment outcome has also been answered.

## Conclusion

Coercive measures in the form of seclusion, whether or not in combination with forced medication, were applied to two-fifths of admitted patients. The symptomatology of three-quarters of the study cohort had improved at the end of treatment. Adjusted for the influence of diagnostic category, the initial HoNOS score (initial severity of illness) and admission duration, the group without coercion had improvements in HoNOS scores that were nearly two and a half points higher on average than the two groups with coercion, a small effect size. A causal relationship between the use of coercion and less favourable treatment outcome could neither be concluded nor ruled out on the basis of our results.

## Data availability statement

The original contributions presented in the study are included in the article/supplementary materials, further inquiries can be directed to the corresponding author.

## Author contributions

LP was the main author and did the data analyses. KN was director of the clinic and supervised the data collection. JP supervised the data analysis. UN participated in the data collection and co-edited the text. JD had the over all supervision of the research. All authors contributed to the article and approved the submitted version.

## Footnotes

^1^The admissions were made under the Dutch Act on Special Admissions to Psychiatric Hospitals (Dutch acronym: BOPZ), which distinguishes between two types of involuntary admission. The first category consists of emergency involuntary admissions (Dutch acronym: IBS): a mayor, advised by an independent physician, decides whether a hospital admission is required to address the emergency situation. The second category (Dutch acronym: RM) is the usual procedure in which a judge determines, on the basis of a medical report from an independent psychiatrist, whether the relevant legal conditions have been met.
